# Effect of melatonin supplemented at the light or dark period on recovery of sciatic nerve injury in rats

**DOI:** 10.17179/excli2016-763

**Published:** 2017-03-06

**Authors:** Enas Ezzat Rateb, Shaimaa Nasr Amin, Nashwa El-Tablawy, Laila Ahmed Rashed, Samah El-Attar

**Affiliations:** 1 Department of Physiology, Beni Swif Faculty of Medicine, Egypt; 2 Department of Physiology, Kasr Al Ainy Faculty of Medicine, Cairo University, Egypt; 3 Department of Biochemistry, Kasr Al Ainy Faculty of Medicine, Cairo University, Egypt

**Keywords:** melatonin, nerve injury, SFI, NCV

## Abstract

Peripheral nerve injuries can cause disabilities, social or economic problems. Melatonin, the secretory product of the pineal gland has antioxidant and anti-inflammatory actions. The aim of the present study was to investigate the effect of melatonin on the recovery of sciatic nerve after injury, comparing its effect when given in the light or the dark periods. Forty adult male Albino rats were allocated into four groups: control, nerve injury, nerve injury + melatonin given at light and nerve injury + melatonin given at dark. Nerve injury was initiated by clamping the sciatic nerve. Sciatic functional index (SFI) was measured preoperatively and postoperatively. Melatonin was given daily for six weeks. Recovery of the function was analyzed by functional analysis, electrophysiological analysis and biochemical measurement of Superoxide dismutase (SOD), Interleukin 1-beta (IL-1 β), Nerve growth factor (NGF), and bcl-2. Melatonin improved SFI, nerve conduction velocity (NCV) and the force of gastrocnemius muscle contraction as compared to the untreated rats. SOD activity, NGF, and bcl-2 were significantly increased, while IL-1β was significantly decreased after melatonin treatment as compared to the untreated injury group. SFI reached the control level; muscle contraction and IL-1B were significantly improved in the group treated with melatonin in the dark. Melatonin fastened the neural recovery and may be used in the treatment of nerve injury and it induced better nerve regeneration when the rats were treated during the dark period.

## Introduction

Peripheral nerves are the most important structures for providing motor and sensory function. The peripheral nerve trauma remains a major cause of motor disability and even with optimal surgical repair; clinically the outcome continues to be very poor (Khan et al., 2014[16]).

When damage occurs to a peripheral nerve, both ischemic and inflammatory processes begin. As part of this process, free oxygen radicals and many toxic agents accumulate around the site of injury resulting in cell destruction with the destruction of neurofilaments and microtubules. Many studies have been performed on surgical techniques; however, studies on medications that might positively affect peripheral nerve regeneration are rare (Atik et al., 2011[2]).

Melatonin (N-acetyl-5-methoxytryptamine) is secreted from the pineal gland, and its secretion is controlled by the circadian rhythm with the blood level being high during the dark period and low during the light period. It is both a lipo- and hydrophilic molecule, and it crosses all physical barriers including the blood-brain barrier and plasma membrane (Zencirci et al., 2010[50]). It is a principal agent which has antioxidant, antiapoptotic, anti-inflammatory (Reiter et al., 2007[34]), and anticarcinogen properties for biological systems (Anisimov et al., 2006[1]).

Melatonin is notified that has neuroprotective effect by reducing nitric oxide syntheses or scavenging free radicals' destructive effects in the nervous system (Anisimov et al., 2006[1]). Also, melatonin has been shown to be effective in arresting neurodegenerative phenomena seen in experimental models of Alzheimer's disease, Parkinsonism and ischemic stroke (Mayo et al., 2005[23]; Poeggeler et al., 2008[32]).

According to the fore mentioned information, we hypothesized a potential beneficial role of melatonin in the treatment of peripheral nerve injury. We aimed at comparing the therapeutic effects of melatonin when given during the light and the dark period. We also hypothesized that such treatment could be partially based on reversing the mechanisms associated with the development of nerve injury like oxidative stress, inflammatory process, and apoptosis.

## Materials and Methods

### Experimental design

The experimental procedures, animal handling, sampling, and scarification were done according to the “Guide for the care and use of laboratory animals” (NRC, 2011[28]) and were approved by Ethical Committee of Physiology Department, Faculty of Medicine, Cairo University. 

40 adult male Wistar Albino rats, 3-4 months old weighing 150 to 200 g constituted the animal model for this study. The animals were housed one per cage (30×15×15cm) with free access to food and water.

Animals were kept under constant laboratory conditions (e.g., 18 °C to 21 °C room temperature, humidity, and illumination of 12 h cycles of light and darkness; light on at 5:00 a.m., light off at 5:00 p.m.).

Animals were randomly allocated into the following four groups (10 rats/group):

Group I [Control group]. These rats were sham operated. They underwent surgical incision without sciatic nerve compression and were given the vehicle. 

Group II [The sciatic nerve injury (SNI) plus vehicle group]: The rats of this group received an intraperitoneal (i.p) injection of the vehicle as a placebo after induction of the nerve injury. 

Group III [The SNI plus melatonin at light group]: In this group, sciatic nerve injury was performed and then the rats received melatonin (Sigma, USA), 50 mg/kg i.p, in the light period (Kaya et al., 2013[15]) at 9:00 am daily for 6 weeks.

GroupIV [The SNI plus melatonin at dark group]: In this group, sciatic nerve injury was performed and then the rats received melatonin, 50 mg/kg i.p in the dark period (Kaya et al., 2013[15]) at 5:00 pm daily for six weeks.

### Surgical technique

Under general anesthesia with xylazine (5 mg/kg) ketamine (50 mg/kg) (Struck et al., 2011[45]), unilateral exposure of the left sciatic nerve at the upper border of the quadratus femoris is done for all the groups. An acute blunt injury was initiated by clamping the sciatic nerves with a standard temporary aneurysmal clip for 2 minutes for all groups except the control one. Next, the clip was removed, the skin incision was sutured, and the animals were returned to their cages (Genovese et al., 2005[12]).

### Procedure of the work

Before performing the nerve injury operation, the rats were tested for the sciatic functional index (SFI). Immediately after the procedure, the rats were tested again, and SFI was measured the 1^st^ day after the operation and weekly throughout the period of the experiment. 

After the experimental period of six weeks, the rats were tested on the walking track; then the rats were anesthetized with xylazine (5 mg/kg) ketamine (50 mg/kg) (Struck et al., 2011[45]). Sciatic nerve and gastrocnemius muscle were exposed then NCV was measured in vivo by PowerLab, and the force of gastrocnemius muscle was measured by Intercept TSC286/1, also in vivo, immediately after measurement of NCV. 

Blood samples were taken from retro-orbital plexus by capillary tube and left to be clotted then centrifuged, and serum was separated and kept frozen for analysis of Interleukin 1-beta (IL-1 β). Rats were then sacrificed, and sciatic nerves were excised from their roots to the beginning of their branches. Each nerve was homogenized in phosphate buffer saline and was kept at -80 °C and was used for measurement of Superoxide Dismutase (SOD) activity, bcl2 expression, and Nerve growth factor (NGF) level.

### Footprint recording and analysis

Preoperatively and continuing weekly throughout the duration of the study, functional recovery was assessed by walking track analysis. For SFI, animals were tested in a confined walkway measuring 100 cm long and 7.5 cm wide with side wall height 15 cm (Kaya et al., 2013[15]). The hind paws of the rats were pressed down onto an inkpad, and they were allowed to walk along a sheet of white paper. The distances between the third toe and heel (Print Length "PL"), first and fifth toe (Toe Spread "TS") and second and fourth toe (Intermediate Toe Spread "ITS") were measured. For crushed leg, it was named as (Experimental Print Length "EPL", Experimental Toe Spread "ETS" and Experimental Intermediate Toe Spread "EITS") and for the control leg (Normal Print Length "NPL", Normal Toe Spread "NTS" and Normal Intermediate Toe Spread "NITS") respectively.

The mean distances of three measurements were used to calculate the following factors:

- Toe spread factor (TSF): (ETS − NTS)/ NTS
- Intermediate toe spread factor (ITSF): (EITS − NITS)/NITS
- Print length factor (PLF): (EPL − NPL)/ NPL
- SFI values were calculated by using the multiple linear regression formulae improved by as follows:
- SFI = −38.3 (PLF) + 109.5 (TSF) + 13.3 (ITSF) − 8.8; where an SFI of 0 represents normal function, and an SFI of -100 demonstrates complete functional loss (Monte-Raso et al., 2008[24]).

### Nerve conduction velocity and force of muscle contraction

Electrophysiological evaluation of nerve regeneration was performed in vivo at the end of the treatment under general anesthesia (Struck et al., 2011[45]). Animals were anesthetized; nerve conduction was measured by PowerLab (Yarar et al., 2015[48]) in vivo after exposure of sciatic nerve (which is mixed nerve with type A fibers).

The force of contraction of gastrocnemius muscle was measured after six weeks, the left limb was fixed, skin opened, and after exposure of sciatic nerve, the distal tendon of gastrocnemius muscle was sectioned and attached to a force transducer to induce isometric contraction (Intercept TSC286/1). The output signals were then fed to a bipotential amplifier (Intercept) and displayed using personal computer-based data capture and acquisition software (Phys 4 intercept). 

The muscle was activated with electrodes stimulating the nerve using monophasic rectangular pulses of a 0.2-millisecond duration of the anodal current. Adjustments to muscle length were made to find the optimal length for maximum twitch force. Stimulus intensity was increased until maximum twitch force was obtained. The Twitch Tension (TT) (expressed as screen units) was recorded Using screen cursors from each gastrocnemius muscle (Chen and Always, 2000[7]).

### Biochemical analysis

#### Sample preparation

60 mg of the sciatic nerve was homogenized in phosphate buffer saline then centrifuged at 10000 rpm for 20 min then the supernatant was kept frozen at -80 °C until used for subsequent analysis of SOD activity, bcl2 gene expression, and NGF level.

The assay was based on the ability of SOD to inhibit the auto-oxidation of epinephrine at alkaline pH. The mitochondrial suspension (0.2 ml) was treated with 0.8 ml of 50 mmol/ l glycine buffer (pH 10.4) and 0.020 ml epinephrine. SOD activity was measured kinetically at 480 nm. The activity was measured indirectly by the oxidized product of epinephrine. MnSOD activity was expressed as nanomoles of (-) epinephrine protected from oxidation per minute per milligram protein by using a molar extinction coefficient of 4020M-1 cm-1 (Nishikimi et al., 1972[27]). 

Detection of bcl2 gene expression was performed by Real time-Polymerase Chain Reaction (real time-PCR):

#### a. Total RNA extraction

Total RNA was extracted from tissues using TRIzol method according to the manufacturer's protocol. In brief, RNA was extracted by homogenization in TRIzol reagent (Invitrogen, Life Technologies, USA). The homogenate was then incubated for 5 min at room temperature. A 1:5 volume of chloroform was added, and the tube was vortexed and centrifuged at 12 000 g for 15 min.

The aqueous phase was isolated, and the total RNA was precipitated with absolute ethanol. After centrifugation and washing, the total RNA was finally eluted in 20 μL of the RNase-free water. The RNA concentrations and purity were measured with an ultraviolet spectrophotometer.

#### b. Complementary DNA (cDNA) synthesis 

The cDNA was synthesized from 1 μg RNA using SuperScript III First-Strand Synthesis System as described in the manufacturer's protocol (Invitrogen, Life Technologies). In brief, one μg of total RNA was mixed with 50 μM oligo (dT)20, 50 ng/μL random primers, and ten mM dNTP mix in a total volume of 10 μL. The mixture was incubated at 56 °C for 5 min, then placed on ice for 3 min. The reverse transcriptase master mix containing 2 μL of 10× RT buffer, 4 μL of 25 mM MgCl2, 2 μL of 0.1 M DTT, and 1 μL of SuperScript® III RT (200 U/μL) was added to the mixture and was incubated at 25 °C for 10 min followed by 50 min at 50 °C.

#### c. Real-time quantitative PCR 

The relative abundance of mRNA species was assessed using the SYBR Green method on an ABI prism 7500 sequence detector system (Applied Biosystems, Foster City, CA). PCR primers (shown in Table 1(Tab. 1)) were designed with Gene Runner Software (Hasting Software, Inc., Hasting, NY) from RNA sequences from GenBank. All primer sets had a calculated annealing temperature of 60 °C. Quantitative RT-PCR was performed in a 25-μl reaction volume consisting of 2X SYBR Green PCR Master Mix (Applied Biosystems), 900 nM of each primer and 2-3 μl of cDNA. Amplification conditions were 2 min at 50 °C, 10 min at 95 °C and 40 cycles of denaturation for 15 s and annealing/extension at 60 °C for 10 min. Data from real-time assays were calculated using the v1·7 Sequence Detection Software from PE Biosystems (Foster City, CA). Relative expression of studied gene mRNA was calculated using the comparative Ct method. All values were normalized to the beta actin gene and reported as fold change over background levels detected in diseases group (Livak and Schmittgen, 2001[20]).

Analysis of serum IL-1β performed by ELISA kit (R&D system, USA) according to manufacturer instructions. 

NGF Level in Sciatic Nerve Tissue measured using Boster's rat NGF ELISA Kit is based on standard sandwich enzyme-linked immune sorbent assay technology (Sanico et al., 2000[40]) according to manufacturer instructions.

### Statistical methods

Data were coded and entered using the statistical package SPSS version 22. Data was summarized using mean ± standard deviation. Comparisons between groups were made using analysis of variance (ANOVA) with multiple comparisons Bonferroni post hoc test in normally distributed quantitative variables while non-parametric Kruskal-Wallis test and Mann-Whitney test were used for non-normally distributed quantitative variables (Chan, 2003[4]). P-values less than 0.05 were considered as statistically significant (Chan, 2003[4]).

## Results

### Results of SFI

Motor performance of the studied groups in the walking track represented by SFI is shown in Table 2(Tab. 2): in the untreated nerve injury group, a significant (P ≤ 0.05) impairment in the motor function was manifested since the 1^st^ day after the injury. This impairment continued throughout the time of the experiment and showed some gradual improvement throughout the experiment but remained significantly (P ≤ 0.05) impaired as compared to the control group till the end of the 6^th^ week.

There wasn't any significant (P ≤ 0.05) difference between melatonin light and untreated nerve injury group till the 1^st^ week. From the 2^nd^ week to the 6^th^ one, there was significant (P ≤ 0.05) improvement of SFI in the melatonin light group (except 3^rd^, 4th weeks) as compared to the untreated nerve injury group. 

There wasn't any significant (P ≤ 0.05) difference between the melatonin dark and the untreated nerve injury group till the 1^st^ week as compared to the untreated nerve injury group. But from the 2^nd^ week to the 6^th^ one there was significant (P ≤ 0.05) improvement of SFI in the melatonin dark group (except 3^rd ^week) as compared to the untreated nerve injury group. There wasn't any significant (P ≤ 0.05) difference between melatonin light and melatonin dark groups throughout the six weeks.

### Results of nerve conduction velocity and force of muscle contraction

**Figure 1:**
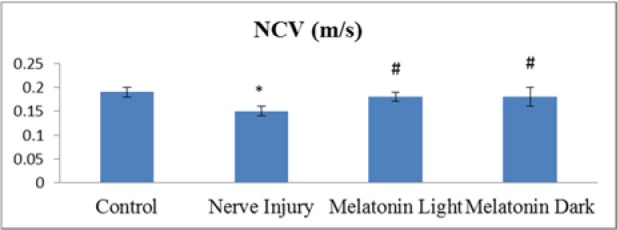
Comparison of NCV between control, nerve injury, melatonin light and melatonin dark groups at the end 6th week of the experiment. Values expressed as mean ± SD. *: statistically significant compared to the corresponding value in the control group, #: statistically significant compared to the corresponding value in the nerve injury group; $: statistically significant compared to the corresponding value in the melatonin light group at P ≤ 0.05.

Recording of nerve conduction velocity in the studied groups (Figure 1(Fig. 1)) reveals that: induction of sciatic nerve injury caused a significant (P ≤ 0.05) impairment in NCV as compared to the control. The significant (P ≤ 0.05) impairment continued throughout the time of the experiment. Melatonin given at the light period enhanced the recovery with a significant (P ≤ 0.05) increase in NCV compared to untreated nerve injury group. Melatonin dark group was improved as there was a significant (P ≤ 0.05) increase in NCV values as compared to the untreated nerve injury group and this improvement approximated the control value. There wasn't any significant (P ≤ 0.05) difference between melatonin light and melatonin dark groups.

**Figure 2:**
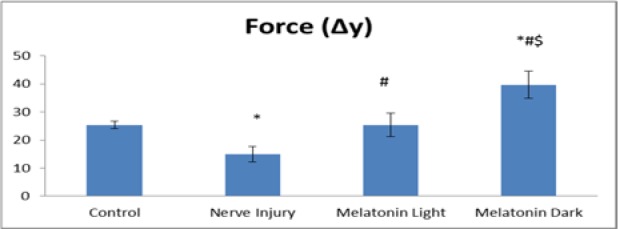
Comparison of muscle contraction between control, nerve injury, melatonin light and melatonin dark groups at the end 6th week of the experiment. Values expressed as mean ± SD,*: statistically significant compared to the corresponding value in the control group, #: statistically significant compared to the corresponding value in the nerve injury group, $: statistically significant compared to the corresponding value in the melatonin light group at P ≤ 0.05.

Induction of nerve injury caused a significant (P ≤ 0.05) decrease in the force of contraction of Gastrocnemius muscle in the untreated nerve injury group, and this significant decrease continued throughout the time of the experiment compared to the control group. There was significant (P ≤ 0.05) improvement of the force of muscle contraction in the melatonin light group compared to nerve injury group and this improvement approximated the control value. In the melatonin, dark group the force of gastrocnemius muscle contraction was highly improved, and its mean value significantly (P ≤ 0.05) increased as compared to control and there was significant (P ≤ 0.05) improvement of the force of muscle contraction as compared to the untreated nerve injury group (Figure 2(Fig. 2)). 

### Biochemical results

Evaluation of biochemical markers in the studied groups (Table 3(Tab. 3)) shows that:

- Induction of nerve injury caused significant impairment in SOD activity as compared to the control throughout the time of the experiment. In the melatonin light group, there was significant (P ≤ 0.05) improvement of its activity as compared to the untreated nerve injury group. In the melatonin dark group, there was a significant (P ≤ 0.05) improvement of SOD activity compared to the untreated nerve injury group. There wasn't any significant difference between melatonin light and melatonin dark groups as regards SOD activity.
- There was a significant (P ≤ 0.05) increase in the serum IL-1β level after the end of the 6^th^ week and the mean for the untreated nerve injury group as compared to the control. In melatonin light group there was a significant (P ≤ 0.05) decrease in the serum level of IL-1β as compared to the untreated nerve injury group. Also; in the melatonin dark group, there was a significant (P ≤ 0.05) decrease in the serum level of IL-1β as compared to the untreated nerve injury group.
- The apoptotic regulator bcl2 gene expression was significantly (P ≤ 0.05) decreased in the untreated nerve injury as compared to the control. Melatonin treatment at light caused a significant increase in bcl-2 expression as compared to the untreated nerve injury group. Furthermore; in the group treated with melatonin at dark, there was a significant (P ≤ 0.05) increase of bcl-2 expression as compared to the untreated nerve injury group. There wasn't any significant (P ≤ 0.05) difference between melatonin light and melatonin dark group.
- There was a significant (P ≤ 0.05) increase in NGF level in the injured sciatic nerve as compared to the control at the 6^th^ week as compared to the control. Melatonin treatment during both light and dark periods caused significant (P ≤ 0.05) increase of NGF compared to untreated nerve injury group. There wasn't any significant (P ≤ 0.05) difference between melatonin light and melatonin dark group.

## Discussion

### SFI in untreated and melatonin-treated groups

Analysis of rat walking tracks by SFI has proven to be a reliable, repeatable, economical, and quantitative method of evaluating function following sciatic nerve injury and repair. Motor function, mainly measured by the SFI can directly reflect nerve function following peripheral nerve injury (Pan et al., 2009[29]).

The results of the present study showed that there was significant impairment in SFI since the 1^st^ day after the injury and continued throughout the time of the experiment. This finding is in agreement with previous studies (Galloway et al., 2005[11]; Sozcukler et al., 2013[44]; Khan et al., 2014[16]).

The effect of nerve injury on SFI was secondary to the loss of ankle plantar flexion, foot inverters, toe flexors, and foot intrinsics, so the footprint characteristically demonstrates an increased print length, a decreased toe spread, and a decreased intermediate toe spread (Sarikcioglu et al., 2009[41]).

In the current study; melatonin treatment significantly accelerated the recovery process in the melatonin-treated groups as compared to the vehicle-treated group (nerve injury) group. The group of rats treated with melatonin at the dark period showed a significantly better performance in SFI test compared to that treated during the light period.

In agreement with our results; studies conducted by Zencirci et al. (2010[50]), and Atik et al. (2011[2]) reporting that the SFI value was increased significantly in the melatonin-treated groups as compared to the untreated group of rats with peripheral nerve crush injury. Improvement of SFI indicates better motor and sensory function revealing nerve and muscle regeneration as suggested by Sarikcioglu et al. (2009[41]). 

These beneficial effects of melatonin administration on SFI effects may be attributed to its antioxidant and anti-inflammatory properties improving the neural recovery after the injury (Kaya et al., 2013[14]). 

### Nerve conduction velocity and force of muscle contraction in untreated and melatonin-treated groups

Nerve conduction, the primary function of peripheral nerves, can be directly reflected by the conduction velocity of the electrical activity of the neural stem (Yang et al., 2007[47]). The results of the present study showed that nerve injury significantly decreased NCV, this by Dai et al. (2015[8]), and Yarar et al. (2015[48]), who found decreased NCV eight weeks after induction of nerve injury.

It was reported that the myelin sheath is a rich source of lipids and is the main target of free radical-mediated lipid peroxidation during trauma causing not only damage to the phospholipids of the neural membranes but also makes myelin proteins more susceptible to the attack of reactive oxygen species (Shokouhi et al., 2008[43]). 

Moreover, the findings of the present study showed that the force of muscle contraction was significantly decreased after nerve injury as compared to the control. These results were agreed by Maciel et al. (2013[21]), and Zhao et al. (2012[51]). On the other hand, Teodori et al. (2011[46]), suggested preservation of muscle contraction after rat sciatic nerve crush between the 10th and 15th day after the injury.

Denervated muscles undergo progressive degeneration, which leads to loss of muscle fibers and their replacement with fat and fibrous connective tissue and this decreases the force of its contraction (Rochkind et al., 2009[37]). Also, with prolonged denervation, the motor end plate area gradually reduced leading to a reduction of the force of muscle contraction (Zhao et al., 2012[51]).

In the current work, melatonin treatment for six weeks induced a significant increase of NCV in the melatonin-treated groups as compared to the untreated nerve injury group, revealing a beneficial effect of melatonin treatment. The increased NCV in the melatonin-treated groups reached approximately the values recorded in the control group, with no significant difference shown between the melatonin-treated groups and the control group.

In accordance with our results, Atik et al. (2011[2]) reported that melatonin treatment (10 mg/kg for 21 days) significantly increased NCV compared with the untreated group.

It appears that melatonin helps development and maturation of myelin sheath of the regenerated nerve or protects it from peroxidation and limits its damage (Shokouhi et al., 2008[43]).

Moreover, the results of the present study showed that melatonin treatment increased the force of muscle contraction significantly as compared to the untreated nerve injury group to return approximately as the mean values recorded in the control group. The group treated with melatonin at the dark period showed a significant increase in muscle contraction force as compared to that treated at the light period revealing the beneficial effect of melatonin treatment especially when given at the dark period.

This finding was consistent with Chang et al. (2014[5]), who induced an in vivo model of peripheral nerve injury and administered Melatonin for 30 days. The proliferative effects of melatonin on Schwann cells are closely related to the improved nerve recovery, increasing the number of reinnervated motor end plates on target muscles following PNI leading to increasing the force of muscle contraction (Chen et al., 2007[6]).

Interestingly, Nassar et al. (2007[26]) showed that taking up to 5 milligrams of melatonin before sleep or during wakeful hours results in increased blood growth-hormone levels. This observation supports our results that the group treated with melatonin at the dark showed a significant increase in muscle contraction as compared to control forming a link between blood melatonin levels and GH release.

### Biochemical markers in untreated and melatonin-treated groups

The observations of the present study also showed that nerve injury decreased SOD activity in the injured nerve tissue as compared to the control group indicating that crush injury to the sciatic nerve causes oxidative stress. These results were in agreement with previous studies on peripheral nerve injury (Atilgan et al., 2014[3]; Kucu et al., 2015[19]).

Radical-scavenging antioxidants such as SOD which is concerned with the removal of superoxide anion and peroxide are consumed by the increased free radical activity. Oxidative stress increases after nerve injury and may be a mechanism of it (Senoglu et al., 2009[42]). 

Moreover, the present work showed that nerve injury induced a significant increase in serum IL-1β level as compared to the control indicating that nerve injury leads to activation of an inflammatory response. This result was in agreement with Ellis and Bennett (2013[9]) and Ydens et al. (2012[49]) 

The first cells to react to damage of the nerve are Schwann cells and resident immune cells. An unspecified signal from damaged axons results in activation of the extracellular signal-related mitogen-activated protein kinase signaling pathway; triggering the expression of inflammatory mediators and recruiting immune cells to the damaged nerve (Napoli et al., 2012[25]). These inflammatory mediators control phospholipase-A2 activation in Schwann cells which is responsible for initial degradation of myelin (Martini et al., 2008[22]). 

Perrin et al. (2005[31]), reported that the administration of a function-blocking antibody against IL-1β into the injured mouse sciatic nerve prevented a reduction in the recruitment of macrophages and retarded myelin phagocytosis. 

Furthermore; the findings of the present study showed that the expression of bcl2 in the injured sciatic nerve significantly decreased as compared to the control. Kotulska et al. (2005[18]) suggested that the expression of bcl-2 is closely related to the recovery and viability of neurons after peripheral nerve injury as well as fiber regeneration and myelination.

The results of the present study also showed that nerve injury increased NGF level in the nerve tissue as compared to control. This finding was in agreement with a study by Richner et al. (2014[35]) who confirmed that the levels of NGF exhibit a biphasic response to axotomy with an immediate early rise in NGF peaking six h after injury. Following nerve injury, a large quantity of production of NGF by fibroblasts, Schwann cells, and macrophages, is triggered by cytokines released from endogenous or exogenous phagocytes (Jing et al., 2009[13]).

The observations of the present study proved that melatonin, administration either at the light or dark periods, improved or fastened recovery of nerve injury. Trying to explain the underlying mechanisms for this beneficial effect, we evaluated the antioxidant activity of melatonin through measuring and comparing SOD activity in nerve tissue in the different groups. We demonstrated that melatonin administration significantly increased SOD activity in the melatonin-treated groups compared to untreated nerve injury group. There was no significant difference between the group of rats treated with melatonin during the dark period and that treated during the light period. These results were in agreement with those reported by Kaya et al. (2013[14]), who reported that melatonin treatment elevated SOD activity in sciatic nerve samples in rats after both cut and crush injury.

Melatonin has profound antioxidant actions against oxidative and nitrosative stress (Esposito and Cuzzocrea, 2010[10]), and is highly effective in scavenging reactive oxygen and nitrogen species. Moreover, melatonin is known to be a stabilizer of cell and organelle membranes because of its inhibitory effects on lipid peroxidation as explained by Reiter et al. (2001[33]). 

Another mechanism explaining the protective effect of melatonin and its potential role in accelerating the recovery after nerve injury is through reducing the inflammatory response associated with the nerve injury. The results of the present study showed that melatonin significantly decreased IL-1β level in serum as compared to the untreated nerve injury group. The group treated with melatonin at the dark period showed significantly more improvement than that treated at the light period revealing the beneficial effect of melatonin treatment especially when given at the dark period.

Melatonin treatment reduced the elevated levels of proinflammatory cytokines like TNF-α and IL-6, in sciatic nerves of rats. The effect of melatonin on IL-1β has also been evaluated in many other tissues, and it was shown that melatonin decreases IL1β as in brain ischemia induced by blockade of the right middle cerebral artery in aging male Wistar rats (Paredes et al., 2015[30]). 

Moreover, the present work showed that the antiapoptotic marker bcL-2 expression was significantly elevated in the melatonin-treated groups as compared to untreated nerve injury group; however, it was still significantly decreased as compared to the control group. 

The antiapoptotic effect of melatonin was also reported by Paredes et al. (2015[30]) who revealed a significant elevation in the mRNA levels of Bcl-2. Some studies have suggested that melatonin inhibits apoptotic processes via its antioxidant properties as reported by Sainz et al. (2003[39]). Other studies have shown that the anti-apoptotic effects of melatonin are accompanied by modulation of the expression of the Bcl-2 family genes (Rubio et al. (2007[38]). Melatonin treatment because of antioxidative and antiapoptotic properties provide beneficial effects on NGF. Melatonin also increases neurotrophic factors by activating its receptors (MT1 and MT2) (Kong et al. (2008[17]).

### Conclusions and recommendations

From the previous data, it was shown that melatonin is vital in the treatment of nerve injury and helping in neural regeneration by its antioxidant, anti-inflammatory and antiapoptotic actions. There was no significant difference when it was given in the light or dark period except in improvement of muscle contraction and IL-1β which were significantly improved in the dark period. Also, SFI reached the control level in the group treated with melatonin in the dark. These effects may be due to the physiological circadian rhythm of it in the body as it is mainly secreted by the pineal gland in the dark. Melatonin also prevents the adrenal gland from responding to ACTH and inhibit cortisol secretion, so when given in the light, it inhibits cortisol secretion affecting its anti-inflammatory action (Richter et al., 2008[36]). 

We conclude that; treatment of nerve injury with melatonin helped in fastening the recovery of the injured nerves with significant improvement of the functional, electrophysiological and biochemical parameters of them. This improvement of nerve regeneration can be explained by the antioxidant, anti-apoptotic and anti-inflammatory effect of melatonin hormone and its role in enhancing recovery through stimulating NGF. The different time of melatonin administration gave different results in nerve regeneration suggesting that giving it at the dark period was better than giving it during the light period and this may be explained by the physiological circadian rhythm of melatonin as it is naturally secreted in the dark period in the body.

Further studies are recommended with evaluation of more parameters indicating changes in the muscle and sciatic nerve that are not measured in our work to explain the beneficial effect of melatonin on enhanced functional recovery recorded in the group with melatonin treatment at the dark period compared to those treated with the same dose but at the light period. 

## Acknowledgements

We would like to thank technicians at the Department of Physiology Mrs. Afaf and Mrs.Azza for their kind help in work.

## Figures and Tables

**Table 1:**
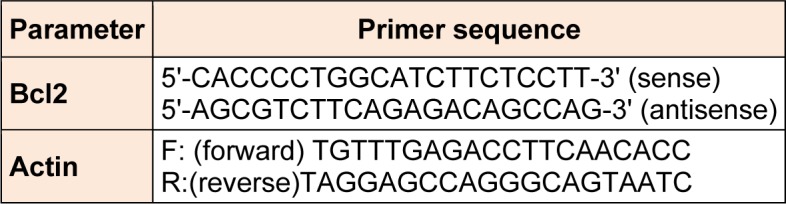
Sequence of the primers used for real-time PCR for bcl-2 measurement

**Table 2:**
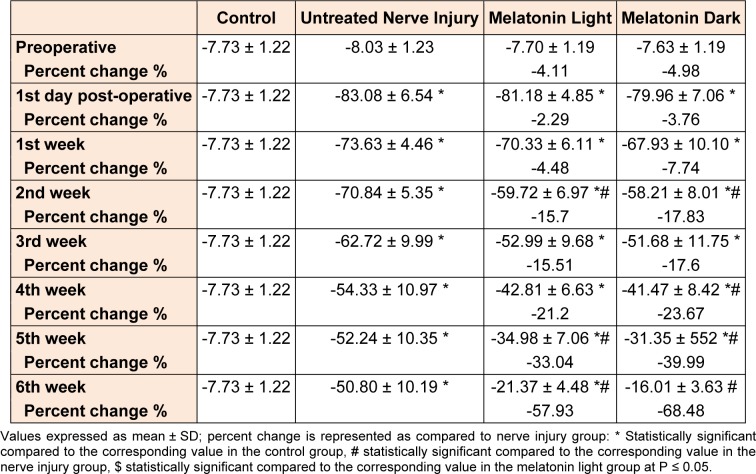
Comparison of SFI between control, nerve injury, melatonin light and melatonin dark groups preoperatively, 1^st^ day postoperative, and throughout six weeks

**Table 3:**
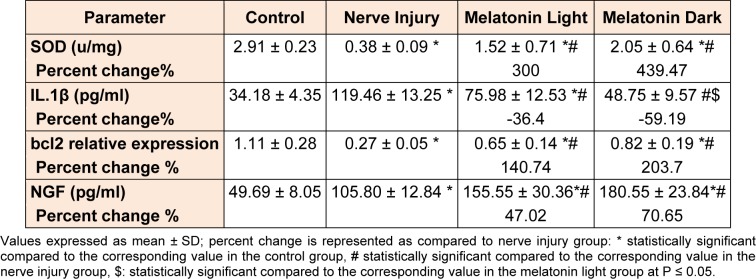
Comparison of biochemical parameters in the studied groups at the end 6th week of the experiment
